# Executive Functions in Tobacco Dependence: Importance of Inhibitory Capacities

**DOI:** 10.1371/journal.pone.0150940

**Published:** 2016-03-08

**Authors:** Valentin Flaudias, Marie Christine Picot, Jorge Lopez-Castroman, Pierre-Michel Llorca, Audrey Schmitt, Jean Perriot, Vera Georgescu, Philippe Courtet, Xavier Quantin, Sébastien Guillaume

**Affiliations:** 1 Clermont Université, Université d'Auvergne, EA NPsy-Sydo, BP 10448, F-63000, Clermont-Ferrand, France; CHU Clermont-Ferrand, Pôle Psychiatrie B, F-63001, Clermont-Ferrand, France; 2 DIM, CHU Montpellier, Montpellier, France; 3 Department of Psychiatry, CHRU Nimes / INSERM U1061, Nimes, France; 4 Dispensaire Émile Roux, Université d'Auvergne, Clermont-Ferrand, France; 5 Department of Emergency Psychiatry and Post-Acute Care, CHRU Montpellier / INSERM U1061, Montpellier, France; 6 Respiratory Disease Department, CHU Montpellier / Laboratoire Epsylon, Montpellier, France; VU University Medical Center, NETHERLANDS

## Abstract

**Background:**

Executive functions are linked to tobacco dependence and craving. In this cross-sectional study, we assessed the impact of three executive functions: updating, inhibition and shifting processes on tobacco craving and dependence.

**Method:**

134 tobacco consumers were included in this study: 81 moderately (Fagerström score <7) and 53 heavily dependent (Fagerström score >7). Dependence was assessed with the Fagerström test and craving with the tobacco craving questionnaire (TCQ 12). We used the Stroop test and the Hayling test to measure inhibition, the Trail Making Test to measure shifting processes and the n-back test to measure updating processes. A multivariate logistic model was used to assess which variables explained best the level of nicotine dependence.

**Results:**

Inhibition (p = 0.002) and updating (p = 0.014) processes, but not shifting processes, were associated with higher tobacco dependence. Inhibition capacity had a significant effect on the nicotine dependence level independently of age, education, time since last cigarette, intellectual quotient, craving, updating and shifting process.

**Conclusions:**

Nicotine dependence level seems better explained by inhibition capacities than by craving and updating effects. The capacity to inhibit our behaviours is a good predictor of the severity of tobacco dependence. Our results suggest a prefrontal cortex dysfunction affecting the inhibitory capacities of heavy tobacco dependent smokers. Further studies are needed to investigate the application of these findings in the treatment of tobacco dependence.

## Introduction

Half of all tobacco users will die from a tobacco-related disease, a total of 6 million deaths or one in every 10 deaths occurring each year in the world [1]. Although smoking cessation programs and public policies against tobacco are being reinforced, especially in industrialised countries, 70% of patients relapse in the first six months after quitting smoking [2]. One reason is probably the strength of nicotine dependence. Indeed, Kandel et al [3] found nicotine more addictive than alcohol, marijuana or cocaine in a large population study in the US (N = 87,915). A better understanding of the underpinnings of nicotine dependence may help to improve smoking cessation programs.

A lot of factors are known to have an impact on smoking dependence: craving [4], age of initiation, level of education [5] and cognitive abilities [6,7]. These abilities concern memory, learning, attention and executive functions (EFs) [8,9]. These last functions could be defined as a set of mental processes that control and regulate other abilities and behaviours. For instance, EFs are needed for goal-directed behaviours and to initiate and stop actions. Miyake et al [10] identified three components of EFs (see also [11], for an alternative review):

1. Updating: the continuous monitoring and quick addition or deletion of contents within one’s working memory. In practice, updating is often interchanged with the concept of working memory [12]. Working memory is a more general concept and updating processes could be seen as a construct of working memory. Given that working memory is probably impaired in smokers compared to non-smokers, the self-medication theory posits that tobacco use could enhance working memory [13–15].
2. Shifting processes: the ability to switch between thinking about two or more different concepts simultaneously [16]. Although this cognitive function has not been specifically explored in smokers, studies with the Trail Making Test (TMT), which measures shifting processes, did not observe any difference between smokers and non-smokers [15,17].
3. Inhibitory processes: ‘the capacity to deliberately inhibit dominant, automatic, or prepotent responses when necessary’ [10]. The impact of inhibition capacities on tobacco dependence is unclear. Some studies did not find differences in inhibitory control between non-smokers and light or heavy smokers [17–19], but other authors have underlined that poor inhibitory control is linked with more dependence [20,21]. Recently, Billieux et al [22] found in non-deprived light-to-moderate smokers that inhibition capacities measured by a go-nogo task had a significant impact on nicotine dependence independent of age, craving and speed processing. Tobacco use could also have an impact on inhibition capacities as measured by a Stroop test or a Continuous Performance Test [23].

A recent study of the neuropsychological factors linked to tobacco use in a large sample (N = 1,002 smokers and 1,161 never smoking controls) found a deficit of visual attention and an excess of impulsivity in smokers compared to non-smokers, but no difference for verbal episodic memory, shifting processes, verbal fluency, verbal working memory and Stroop interference [15]. However, two relevant factors that are likely to influence prefrontal cortex (PFC) dysfunction caused by tobacco dependence were not controlled for: level of dependence and craving.

In this study, we aim to investigate the influence of EFs (shifting, updating and inhibition processes) on the level of dependence of a sample of current smokers, while adjusting for craving. We hypothesise that dependence levels will be associated with updating and inhibition impairments, but not with shifting processes.

## Method

### Study design and participants

This cross-sectional study is an ancillary project of a cohort study aiming to evaluate neurocognitive factors involved in smoking relapse (clinicaltrials.gov NCT01554436). The local research ethics committee approved the study (Comité de protection des personnes ‘Sud-Méditerranée IV’ Lapeyronie Hospital, Montpellier, France) and all participants completed and returned a consent form.

A total of 134 patients (74 women and 60 men) were included. They were consecutively recruited in a tobacco smoking cessation program in two academic centres in France (Montpellier and Clermont-Ferrand university hospitals). All participants were current and non-deprived smokers at the time of assessment and were seeking a smoking cessation program. Inclusion criteria consisted of: 1) being a tobacco consumer (Fagerström > 3), 2) age between 18 and 60, and 3) being a native or fluent French speaker. Pregnant women and hospitalised patients were not included. They only non-inclusion criterion was an inability to be followed for 6 months. Importantly, having a psychiatric disorder was not an exclusion criterion. twenty eligible subjects refused to participate and were not included during this period. There was no significant difference in demographic characteristics between included and non-included subjects.

### Clinical assessment

A senior tobacco specialist collected demographic data, actual medication and smoking characteristics during the inclusion visit. Tobacco dependence and craving were assessed. Previous studies have shown that psychiatric comorbidities have an effect on nicotine dependence, in particular depression [24] and substance use disorders [25]. Thus, we measured comorbidities and in particular alcohol dependence and level of depression. As the acute effect of nicotine on neurocognitive performance is well demonstrated [13], we also measured the time since the last cigarette (TSLC).

#### Psychiatric Comorbidity

Psychiatrics diagnoses were assessed using the *Mini International Neuropsychiatric Interview (MINI)* [26]. We also used the *CAGE test* [27] to assess alcohol use. This is a brief questionnaire (four questions) to assess alcohol use. Patients with a score of at least 2 on this test were diagnosed as presenting ‘alcohol misuse’. Finally, a dimensional assessment of mood status was obtained using the French version of the *Montgomery-Asberg Depression Rating Scale (MADRS)* [28].

#### The Tobacco Craving Questionnaire (TCQ-12)

The TCQ-12 includes 12 self-report questions that evaluate four dimensions of craving: emotionality, expectancy, compulsivity and purposefulness [29]. The α-Cronbach in our population between the twelve items was 0.77, which represents a good reliability for the tool.

#### Fagerström Test for Nicotine Dependence [30]

We used the French version [31] to measure the level of smoking dependence. This self-report questionnaire comprises 6 questions scored from 0 to 1 or 0 to 3, and yields a total score that ranges from 0 (not dependent) to 10 (highest dependence). Two groups of dependent patients, heavy and moderate, were identified according to their score on the Fagerström test. In accordance with the ‘French national consensus conference’ [32], patients with scores equal or greater than 7 were considered heavy smokers.

### Neuropsychological assessment

On the same day, patients underwent the neuropsychological tests with a neuropsychologist.

#### Intellectual functioning: fNART [33]

Premorbid intelligence was assessed using the French language adaptation of the National Adult Reading Test [34]. The test consists of 33 words, graded in difficulty, whose pronunciation cannot be determined from their spelling. A score of correct answers was calculated. This score was proved to be highly correlated with the Wechsler Adult Intelligence Scale score [33]. An estimated intellectual quotient (IQ) could be calculated with the following formula: IQ = 124.44–1.54 x fNART errors [33].

#### Updating processes: n-back test [35]

The n-back measures the updating process of working memory. In this task, squares are briefly presented on a computer screen to the participants. The subject must remember if the current location of the square is the same as the previous square position on rial back (1-back), of the square position two trials back (2-back) and of the square position three trials back (3-back). The number of correct answers was an indicator of updating capacity.

#### Shifting processes: TMT A and B [36] measure shifting processes

On the TMT A the subject is required to draw lines to connect consecutively numbered circles. On the TMT B the subject is required to draw lines to connect consecutively numbered and alphabetised circles alternately. This phase assesses a flexible conceptual shifting while keeping track of the sequences of letters and numbers. Performance is assessed with the time it takes in seconds to complete the two tasks. A lower score indicates a better shifting capacity. The number of errors and the corrected errors are also indicators of performance on this task.

### Inhibition capacities: Hayling test and Stroop test

The Hayling test [37] measures the capacity to inhibit an automatic semantic answer which is appropriate. In the first part of this test, the participant has to complete a sentence with a semantically appropriate word (part A). In the second part of this task, which is the inhibition part, the same sentence has to be completed with a non-semantically related word (part B). Three scores can be calculated: i) initiation, the total time to answer the fifteen sentences of part A (Hayling A); ii) inhibition, the total time to answer the fifteen sentences of part B (Hayling B); and iii) interference, the difference between the scores on the two parts (Hayling B-A). The higher the score the lower the inhibition capacity.

The Stroop word color test proposed by Golden [38], which is derived from the classical Stroop test [39]. In this procedure the subject has 45 seconds to: (a) read colour-related words printed in black ink (Stroop W), (b) name the colour of ‘XXXXs’ printed in colour (Stroop C) and (c) identify the colour of words printed in coloured ink, where colours and words do not match (Stroop WC). The number of correct answers is recorded and an interference score is calculated as follows: WC-[(W x C)/(W + C)]. Where these tasks are concerned, the fNART and n-back were computerised tests, and while the TMT, Hayling test and Stroop test were pencil tests.

### Statistical analyses

Descriptive analyses were performed in the whole sample and in the groups of moderately and heavily dependent subjects. Demographic, clinical characteristics and neuropsychological scores were compared between the two groups using a Mann-Whitney test for quantitative variables. When the difference was significant, Cohen’s d was calculated. Qualitative variables were compared using either a chi-square test or Fisher’s exact test when the chi-square validity conditions were not met.

Pearson’s correlations were computed between neuropsychological performances, clinical and tobacco-related variables. To correct for multiple testing, several adjustment methods were used and provided similar results: the Bonferroni adjustment, the step-down Bonferroni adjustment and the linear step-up adjustment based on the false discovery rate (SAS proc multtest with the options: bonferroni, holm, fdr).

Finally, a multivariate logistic regression was performed to assess which variables best explained the level of nicotine dependence. Explanatory variables were introduced into the model according to their clinical relevance using expert knowledge. To assess the predictive ability of the model, the concordance rate between predicted and observed responses was calculated. Statistical significance was set to 0.05 for all analyses. Analyses were performed with SPSS 19 (IBM, Armonk, NY, USA) and SAS 9 (SAS Institute, Cary, N.C).

## Results

The sample was composed of 81 (36 men and 45 women) moderately dependent smokers and 53 heavily dependent smokers (24 men and 29 women).

### Relationship between demographic, clinical and tobacco-related data with dependence level

Table 1 summarises the characteristics of the whole sample and of both moderately and heavily dependant samples. Heavily dependent smokers presented significantly higher levels of craving (p = 0.016; d = -0.47), and a non-significant trend for less time since last cigarette (p = 0.08). There were twice as many patients on antidepressants in the high dependence group than in the moderate dependence group (p = 0.02). There were no other differences between the groups in terms of age, level of education, sex, level of alcohol dependence, level of depression, and intellectual quotient.

**Table 1:** Demographic and clinical characteristics of all patients and with moderate and heavy dependence.

Patient characteristics	All patients Mean (SD) or n (%)	Moderate dependence Mean (SD) or n (%)	Heavy dependence Mean (SD) or n (%)	p-value Wilcoxon Mann-Whitney test or chi square test^x^ or Fisher test ^f^	Effect size (Cohen’s d)
***n***	134	81	53		
**Age**	47.37 (10.83)	47.33 (11.90)	47.43 (9.06)	0.726	
**Sex**				0.924^x^	
***M***	60 (45%)	36 (44.4%)	24 (45.3%)		
***F***	74 (55%)	45 (55.6%)	29 (54.7%)		
**Education level**				0.207 ^x^	
***No High School Diploma***	47 (35%)	4%	8.3%		
***Beyond High School***	87 (65%)	21.3%	27.1%		
**Current Medication**					
***Antidepressant***	27 (20%)	11 (14%)	16 (30%)	**0.020** ^x^	
***Benzodiazepine***	25 (19%)	14 (17%)	11 (21%)	0.615 ^x^	
***Antipsychotics***	6 (4%)	4 (5%)	2 (4%)	0.751 ^f^	
***Mood stabiliser***	13 (10%)	6 (7%)	7 (13%)	0.269 ^x^	
**Current psychiatric comorbidity**					
***Major depressive disorders***	5 (4%)	3 (4%)	2 (4%)	0.983 ^f^	
***Bipolar disorder***	18 (13%)	13 (16%)	5 (9%)	0.261 ^f^	
***Any anxiety disorders***	38 (28%)	25 (31%)	13 (24%)	0.440 ^x^	
**MADRS**	7.34 (6.87)	7.46 (6.77)	7.17 (7.09)	0.800 ^x^	
**CAGE**	6.79 (1.20)	0.87 (1.23)	0.66 (1.14)	0.269	
**Time since last cigarette (min)**	111.47 (331.89)45 (20–60)*	88.76 (188.39)45 (30–60)*	147.34 (478.35)32.5 (10–60)*	**0.08**	-0.16114
**Craving**	39.12 (13.05)	36.84 (12.30)	42.95 (13.53)	**0.016**	-0.47252
**IQ**	107.56 (8.26)	108.49 (6.84)	106.17 (9.93)	0.361	

MADRS: Montgomery-Asberg Depression Rating Scale; CAGE: score at the CAGE questionnaire; craving: total score of the 12 items of the Tobacco craving Questionnaire; IQ: Intellectual quotient assessed by fNART.

* median (1st quartile– 3rd quartile)

### Relationship between neuropsychological performances and dependence level

In the univariate analysis, no significant differences were observed between moderately and heavily dependents concerning IQ total and shifting processes (TMT; Table 2). Concerning updating, higher n-back total scores were observed among moderate dependents (p = 0.014), a group effect for Hayling part A (p = 0.021), part B (p<0.001), and Hayling B-A (p = 0.002) showed that heavily dependent smokers had poorer inhibition capacities than moderately dependents. None of the Stroop test scores were significantly different between groups.

**Table 2:** Comparison of level of dependence with regards to the scores of neuropsychological tests.

Domain	Variable	All patients Mean (SD)	Moderate dependence Mean (SD)	Heavy dependence Mean (SD)	p-value (Wilcoxon Mann-Whitney test)
Intellectual functioning	IQ Total	107.6 (8.26)	108.49 (6.84)	106.17 (9.93)	0.36
Updating process	1-back tot	44.47 (3.98)	44.25 (5.09)	47.49 (0.78)	0.99
	2-back tot	39.84 (5.99)	40.35 (6.24)	39.04 (5.54)	**0.012**
	3-back tot	32.32 (7.03)	32.92 (7.58)	31.40 (6.06)	0.097
	n-back tot	116.14 (15.24)	118.49 (11.12)	112.59 (19.51)	**0.014**
Shifting processes	TMTA RT	34.01 (10.58)	33.53 (10.87)	34.75 (10.20)	0.39
	TMTA CE	0.25 (0.51)	0.27 (0.55)	0.21 (0.46)	0.67
	TMTA NCE	0.04 (0.23)	0.05 (0.27)	0.02 (0.14)	0.53
	TMTB RT	91.31 (91.71)	91.31 (113.37)	91.31 (43.21)	0.054
	TMTB CE	0.32 (0.66)	0.23 (0.60)	0.44 (0.73)	**0.037**
	TMTB NCE	0.36 (1.72)	0.32 (1.92)	0.42 (1.41)	0.19
Initiation	Hayling A	531.41 (394.08)	483.55 (381.99)	604.55 (404.60)	**0.021**
Inhibition	Hayling B	3,740.92 (4916.04)	2,851.64 (2079.53)	5,128.19 (7253.95)	**<0.001**
	Hayling B-A	3,191.09 (4885.73)	2,351.75 (1937.10)	4,500.44 (7287.01)	**0.002**
	Stroop W	100.46 (15.21)	100.95 (15.74)	99.73 (14.94)	0.66
	Stroop WC	40.87 (11.22)	41.72 (12.43)	39.61 (9.12)	0.30
	Stroop inter.	-0.45 (8.83)	0.33 (9.38)	-1.60 (7.89)	0.23

Tot: Total; RT: response time; CE: corrected errors; NCE: not corrected errors; inter: interference.

We further explored the relationship between neuropsychological performances and the clinical features either associated with dependence in our univariate analysis or known to potentially impact neuropsychological outcomes. There were two significant correlations after correcting for multiple testing. A negative correlation (-0.35; Bonferroni p value of 0.003) was observed between the Stroop interference score measuring inhibition and the TMTB RT score measuring shifting process and a positive correlation was found between IQ and updating process (0.33; Bonferroni p value of 0.009).

No correlation between neuropsychological performances and craving score, time since last cigarette, MADRS score were observed.

### Multivariate analysis to explain the level of nicotine dependence

Finally, in order to explore the specific impact of each variable on dependence, all clinically relevant variables were introduced into the model: demographic variables (age, education), tobacco-related variables (time since last cigarette, craving), clinical variables (antidepressant, IQ) and neuropsychological performances (updating process (n-back tot), shifting process (TMTB RT) and inhibition process (Hayling B-A, Stroop interference)). We found that only inhibition capacities (Hayling B-A) significantly affected the level of dependence (p = 0.0138, concordance rate 73.4%).

## Discussion

The aim of this study was to explore the link between executive functions and nicotine dependence considering level of craving and time since last cigarette. Thus, we compared cognitive processes (updating, shifting and inhibition) between moderately and heavily dependent smokers. Our results suggest that nicotine dependence level seems better explained by inhibition capacities assessed by the Hayling test than by craving and updating effects. In line with previous studies [15,17], we did not observe any effect for shifting processes.

Although heavily dependent smokers presented higher levels of craving compared to moderately dependent smokers, craving was no longer a significant predictor of nicotine dependence once inhibition scores, IQ, education, age, TSLC and other EFs were entered into the model. In other words, the capacity to inhibit seems to modulate the association between tobacco craving and dependence: the more inhibition capacities the less craving and dependence. Inhibition is a common factor of many important components of addiction such as impulsivity [40], attentional bias [41], or compulsive behaviours [8,42]. Specific targeting of inhibition in clinical studies might help to improve knowledge and management of addiction.

Our results are in line with those reported by Billieux et al [22], who associated the lack of inhibitory control with tobacco dependence using a different measure of inhibition capacities (a go-nogo task)[22]. Other studies failed to find differences in inhibitory control between non-smokers and light smokers or heavier smokers but were limited by: i) non-representative samples (only adolescents); ii) small sample sizes (n<35 in any of them); and, iii) unclear definitions, such as a confusion between inhibition capacities and impulsivity or between inhibition ‘impairment’ and ‘difficulties’ [17–19]. Indeed, inhibition can be seen as a cognitive function, as we do, or as a personality dimension that characterises impulsivity. The Whiteside and Lynam [43] model of impulsivity defines the dimension of ‘urgency’ as the ‘inability to inhibiting a dominant response or automatic’. This definition corresponds to the inhibition capacities, which would be thus a part of general impulsivity.

The other problem concerns the use of some cognitive tests, which were initially designed to assess the cognitive deficit in brain-damaged patients. They provide a threshold that delimitates normal functioning and problematic functioning. In studies where we used these tests as a continuous variable assessing the level of cognitive abilities (and not considering a threshold of dysfunction), we observed a relationship between this cognitive ability and dependence. Thus our results did not suggest that all smokers have impaired inhibition capacities but more that dependents show less capacities compared to non-dependent individuals. Studies with larger samples (n = 50 for Billieux et al; n = 282 for Wilson and MacLean [44]; n = 134 for our study), which assessed inhibition capacities and not impairments, observed a link between inhibition capacities and tobacco dependence.

Surprisingly, no interference effect was observed on the Stroop task, which tests inhibition. This result suggests that the inhibition processes measured by the Stroop task and the Hayling test are not equivalent. Following the proposal by Burgess and Shallice [37], we have chosen the Hayling test, because it is probably more appropriate for clinical practice and more sensitive since it measures the capacity to inhibit a correct answer and also the capacity to create an efficient strategy. Furthermore, the two possible answers in the Hayling test are in the same dimension (words) while the Stroop task requires inhibiting a different dimension (e.g. the meaning of the word) in favour of the dimension of interest of the stimulus (e.g. the ink colour of the word).

A major field of research in addictive disorders endeavours to identify distinctive neurobehavioral endophenotypes that predispose individuals to compulsive drug use. Evidences coming from other fields suggests that response inhibition and inhibitory control might be heritable [45–47]. In smoking behaviours, recent data suggest that cognitive deficits in inhibitory control are present before starting the use of tobacco. For instance, prospective studies have shown that inhibitory capacities in young people are predictive of consumption of alcohol or cigarettes in adult life [48,49]. Also, Yakir et al [50] showed that attention and impulsivity were predisposing factors for tobacco dependence. Although we cannot study temporal associations in our data, all these results highlight the interest in assessing if low inhibition abilities might be a heritable marker of vulnerability to tobacco consumption.

Within a brain localisation perspective, substance dependence is known to be associated with the mesolimbic system which originates in the ventral tegmental area and projects to reward-related brain areas such as the nucleus accumbens, the amygdala, the hippocampus and the prefrontal cortex (PFC) [51]. This last brain region and its important role in addiction is the object of a large body of literature [52]. In the case of tobacco dependence, the PFC is essentially described as a brain region that contributes to the development of craving, the response to smoking cues, and the compulsive behaviour of dependence [53,54].

The PFC is also known to be responsible for EF [55]. In this area, our results support a PFC dysfunction in tobacco dependent subjects, particularly a dorsolateral PFC (DLPFC) dysfunction among heavy smokers. Indeed, n-back and Hayling tests are highly linked to this brain localisation [56,57]. A recent review seems to confirm this hypofunction in various regions of the PFC among patients with substance use disorders [7].

Accordingly, functional magnetic resonance imaging studies have shown a differential activation in the amygdala and the PFC during these tasks with and without nicotine consumption [58,59].

Future treatments of dependence could try to enhance the control of automatic answers and inhibition capacities using DLPFC stimulation. New lines of research have recently opened possibilities in this area. For example, cognitive remediation could improve inhibitory capacities [60]. Recent studies have shown that a non-invasive brain stimulation of the DLPFC with repetitive transcranial magnetic stimulation (rTMS) and transcranial direct current stimulation (tDCS) could enhance decision-making and thus, inhibition capacities [61–63]. However, we did not measure directly the localisation of brain dysfunctions. Therefore, our results should be taken cautiously as future studies exploring this cognitive dysfunction and its precise localisation are needed.

Our results should be interpreted with caution. Firstly, craving is known to have many dimensions, which could explain its lack of effect on nicotine dependence after controlling for inhibition capacities. Further studies are needed to explore the impact of inhibitory control on different dimensions of tobacco craving. Secondly, the results of neurocognitive tests do not correspond exactly to a single function. For example, the n-back test assesses not only working memory, but also updating and visuo-spatial processes. Collette and Van der Linden [64] suggested that EFs should be understood as an interplay between different brain regions. Therefore, as none of the neuropsychological tasks is specific to a function and/or a structure, future investigations are needed to understand the processes involved in the executive tasks, and more particularly their link with PFC localisation.

To conclude, our results show the importance of the link between inhibitory capacities, craving and level of dependence. A better understanding of the relation between inhibitory control and important concepts on addiction such as attentional bias, craving, impulsivity and compulsion are needed. More research is warranted to explore the inhibitory capacities of tobacco smokers and their future use as therapeutic targets.

## Supporting Information

S1 Table Underlying data of this study. (XLS)
